# Emerging Pharmacological Treatments for Migraine in the Pediatric Population

**DOI:** 10.3390/life12040536

**Published:** 2022-04-05

**Authors:** Luigi Francesco Iannone, Francesco De Cesaris, Pierangelo Geppetti

**Affiliations:** 1 Section of Clinical Pharmacology and Oncology, Department of Health Sciences, University of Florence, 50121 Florence, Italy; luigifrancesco.iannone@unifi.it; 2 Headache Center and Clinical Pharmacology Unit, Careggi University Hospital, 50134 Florence, Italy; francesco.decesaris@unifi.it

**Keywords:** CGRP, monoclonal antibodies, gepants, childhood, adolescents, migraine, pharmacologic treatments, devices

## Abstract

Headaches in children and adolescents have high incidence and prevalence rates, with consequent elevated disability costs to individuals and the community. Pediatric migraine is a disorder with substantial clinical differences compared to the adult form. Few clinical trials have been performed specifically on primary headache in pediatric populations using acute and preventative treatments, often with conflicting findings. The limited high-quality data on the effectiveness of treatments are also due to the high placebo effect, in terms of reductions in both the frequency and intensity of migraine attacks in the pediatric population. The recent introduction of calcitonin gene-related peptide (CGRP) pathway inhibitors and ditans is changing the treatment of migraine, but the majority of the data are still limited to adulthood. Thus, few drugs have indications for migraine treatment in the pediatric age group, and limited evidence gives guidance as to the choice of pharmacotherapy. Herein, we review the current evidence of pharmacological treatments and ongoing clinical trials on acute and preventative treatments in the pediatric population with migraine.

## 1. Introduction

Headaches in children and adolescents have high incidence and prevalence rates, with consequent elevated costs to individuals and the community [1]. The prevalence of headaches varies considerably approximatively from 5.9 to 88% [2,3] depending on diagnostic criteria and age, reaching a peak at about 11–13 years of age [3] with migraine and tension-type headache as the most predominant forms. The frequency is higher in males before puberty, with an inverse relationship thereafter [4,5]. Pediatric migraine is a potentially disabling disorder that has substantial clinical differences compared to the adult form. The average prevalence of migraine ranges from 8% to 24% in school-aged children [6]; among these, 0.6 to 1.8% of adolescents and 0.6% of children have a diagnosis of chronic migraine (defined as more than 15 days with headache per month, of which there are ≥8 migraine days) [7,8]. The diagnosis of primary headaches in pediatric patients follows the criteria of the International Headache Society (IHS) [9]. Overall, these criteria could have some limitations that apply to the pediatric age, and the latest version (ICHD 3) [9] addresses some specifics of migraine in pediatric age, such as shorter duration (less than 4 h) and unilateral/bilateral localization of pain [10].

Evidence-based support for the treatment of pediatric migraine remains unsatisfactory as not many specific clinical studies on acute and preventative therapies have been carried out at present. Few clinical trials have been designed and performed in pediatric patients, and conflicting results are often reported [11,12]. Furthermore, randomized controlled trials (RCTs) in the pediatric age group studying the efficacy of preventive migraine medications have reported inconsistent results. The high impact of the placebo effect in young migraineurs [13,14], with a great reduction in both the frequency and intensity of migraine attacks in the placebo arm, is probably one of the reasons for the many discrepant RTCs [15]. The placebo effect, although useful in clinical practice, represents an unsurmountable bias in both pharmacological and non-pharmacological interventional studies [16,17]. Recently, the Childhood and Adolescent Migraine Prevention (CHAMP) trial [18] compared the effects of amitriptyline and topiramate against placebo in pediatric migraine. The trial, which was stopped early owing to futility, showed no significant differences in reductions in headache frequency or headache-related disability over a period of 24 weeks, and the active drugs showed higher rates of adverse events [18,19]. These findings could suggest that the adult model of headache treatment may not apply to pediatric patients, considering that these drugs are effective in adults as reported by clinical trials and observational studies [20].

In recent years, significant advancements have been made in the acute and preventive treatment of migraine and cluster headache. The development of small molecules (gepants) and monoclonal antibodies against the proalgesic neuropeptide, the calcitonin gene-related protein (CGRP) and its receptor [20,21], and serotonin receptor 5-HT_1F_ agonists (ditans) [22] has substantially changed migraine patient care. However, RTCs on these new drugs in the pediatric population are ongoing, meaning that evidence-based guidance for these patients is still unavailable. Herein, we summarize the emerging pharmacological treatments for migraine in the pediatric population, focusing on ongoing clinical trials.

## 2. Diagnosis and Current Evidence-Based Management

The ICHD has been highly successful in diagnosing children and adolescents to be enrolled in clinical trials. In the latest version, ICHD-3, notes and comments were used to distinguish the specific features of pediatric migraine, including shorter duration, frontotemporal location, and allowance for parental observation [9,10]. For children and adolescents, the duration of a migraine attack is reduced to 2–72 h, recognizing that children and adolescents may have headaches of a shorter duration. Furthermore, the characteristics of headache in children are not those typical of adults, and therefore it may be necessary to infer the symptoms from the child’s behavior during the attack itself. To establish the diagnosis, a detailed medical history and physical exam should be performed. The family and patient should be provided with an individualized treatment plan for their headaches, inclusive of medications and/or accommodations the child may need to receive at school [10,23].

Practice guidelines on acute and preventative treatments for migraine in children and adolescents have recently been published by the American Academy of Neurology (AAN) [12,24]. They systematically assessed all RCTs that evaluate acute and preventative migraine treatments in children and adolescents for assessing efficacy compared with placebo. However, they excluded non-pharmacologic therapies and nutraceuticals [25]. Outcomes such as headache pain and associated symptom reduction and freedom from headache were used for acute treatments, whereas headache frequency reduction was used for preventative treatments.

### 2.1. Acute Treatments

For the treatment of acute migraine, the recommendations focus on the importance of early treatment, choosing the route of administration best suited to the characteristics of the individual migraine attack and providing counselling on lifestyle factors that can exacerbate migraine, including trigger avoidance and medication overuse [24]. There is evidence to support the efficacy of the use of ibuprofen, acetaminophen (in children and adolescents), and triptans (mainly in adolescents) for the relief of migraine pain, although the evidence varies for each agent. There is high confidence that adolescents receiving oral sumatriptan/naproxen and zolmitriptan nasal spray are more likely to be headache-free at 2 h than those receiving placebo [24].

About one-third of pediatric patients with migraine remain refractory to acute therapies, and prolonged painful migraine sometimes requires emergency department (ED) management with intravenous (IV) treatments. However, the choice of therapy varies widely in different EDs and there is still no consensus regarding the use of various IV treatments in children and adolescents [26]. Common intravenous treatments for migraine in the pediatric population have recently been reviewed [4,26].

### 2.2. Preventative Treatments

Regarding preventative treatment, the above-mentioned CHAMP study [18,19] demonstrated that, contrary to adults, amitriptyline and topiramate do not show a favorable risk–benefit profile for use in pediatric migraine prevention over at least 24 weeks. The AAN guidelines have reported that there is insufficient evidence to determine if children and adolescents receiving divalproex, onabotulinumtoxinA, nimodipine, or flunarizine are more or less likely than those receiving placebo to have a reduction in headache frequency [12]. Children with migraine who receive propranolol could be more likely than those receiving placebo to have a reduction of at least 50% in headache frequency. Overall, those receiving topiramate and cinnarizine are probably more likely than those receiving placebo to have a decrease in headache frequency. Children with migraine receiving amitriptyline plus CBT are more likely than those receiving amitriptyline plus headache education or amitriptyline alone to have a reduction in headache frequency [12].

Although results from RCTs in children and adolescents are not yet available for antibodies or small molecules against the CGRP pathway, the American Headache Society (AHS) has proposed recommendations on the use of these drugs for pediatric headache disorders [27]. The authors suggested that the use of CGRP receptor antagonists could be considered in adolescent patients with frequent migraine attacks (≥8 headache days/month), with moderate to severe disability associated with migraine (evaluated with a PedMIDAS score of ≥30), and who have failed ≥2 preventive therapies [27]. For younger patients who are refractory to multiple preventive therapies, CGRP receptor antagonists may also be considered with proper monitoring (e.g., bone health, linear growth, weight/BMI, and infections) [17,27]. As reported below, several RTCs testing anti-CGRP antibodies and small molecules are ongoing.

## 3. Drugs under Development

Recently, the International Headache Society developed ad hoc guidelines to assist in the design and execution of well-controlled clinical trials of pharmaceuticals, biologics, devices, and behavioral preventative interventions in children and adolescents with migraine [28]. The recommendations include participant features, trial design, outcome measures and endpoints, statistics, and others. Considering the high placebo effect and the inconsistency of the results of several RCTs in pediatric populations [11,15,18], some recommendations are fundamental to properly design trials and perform enrolment. For instance, age at entry must assure adequate age strata, defining children as participants aged 6 to 11 years, and adolescents as participants aged 12 to 17 years. Data on participants younger than 6 years of age should be considered as observational and safety testing. A minimum 28-day prospective baseline period followed by a treatment period of at least 12 weeks is recommended. The authors defined two possible primary efficacy endpoints: change in headache frequency, as measured by headache days or migraine days, and a 50% responder rate, as measured by migraine days [28]. No guidelines for controlled trials with acute treatments have been published to date by IHS. Several clinical trials are currently ongoing in pediatric patients with migraine.

### 3.1. Literature Search Strategy

We searched (January 2022) MEDLINE (accessed by PubMed), the Cochrane Central Register of Controlled Trials (CENTRAL), and the US National Institute of Health Clinical Trials Registry (www.Clinicaltrials.gov [accessed on 22 January 2022]) for active, ongoing trials involving pharmacological treatments in the pediatric population (defined as patients aged <18 years) with migraine. The search terms were (migraine OR chronic migraine OR episodic migraine) AND (children OR adolescents OR pediatric). Therefore, we selected only interventional studies with active status (i.e., excluding completed, suspended, terminated, withdrawn, and unknown status studies), involving pharmacological treatments regardless of recruitment status. Non-pharmacological devices, cognitive behavioral therapy (CBT), and techniques such as acupuncture were excluded. There were no date limitations or language restrictions.

### 3.2. Preventative Treatments

Of 14 active clinical trials, regardless of the phase, 10 (71.4%) involve an mAbs against the CGRP pathway or gepants. Specific study features, including primary endpoint, status, and age range, are reported in Table 1. In recent years, several anti-CGRP drugs have been marketed, including gepants, small molecule CGRP receptor antagonists (i.e., rimegepant, ubrogepant, and atogepant) and CGRP-pathway-targeted mAbs (i.e., erenumab, eptinezumab, fremanezumab, and galcanezumab) [29]. Several clinical trials and real-world observational studies have consistently demonstrated their effectiveness and tolerability for the acute or preventive treatment of migraine in adults [30]. Considering the breakthrough impact of drugs acting on the CGRP pathways in adults, it is expected that the majority of ongoing RCTs in pediatrics involve small molecules or monoclonal antibodies already approved for patients > 18 years.

In particular, three studies (two phase III and one phase I) [31,32,33] are assessing erenumab in episodic and chronic migraine; two phase III studies are investigating galcanezumab in episodic and chronic migraine [34,35]; one phase III study is evaluating fremanezumab [36]; and three studies (two phase III and one phase I) are investigating eptinezumab [37,38,39]. All these studies are active with ongoing recruitment. One phase III study is assessing rimegepant as a preventative drug in episodic migraine, but recruitment has not yet started [40].

**Table 1:** Ongoing studies on preventative treatments in the pediatric population.

Treatments and Comparators	Status	Phase	Diagnosis	Age Range	Primary Endpoint	Planned Patients/Treatment Duration	Trial Number
**1. Alpha lipoic acid 300mg** **2. Flunarizine 5 mg**	Recruiting	IV; open label	Chronic migraine	10 to 19 Years	Mean monthly migraine attack rate	60/12 weeks	NCT04064814 [41]
**Erenumab three doses adjusted by weight**	Recruiting	I; open label	Migraine with or without aura	6 to 17 Years	Serum PK parameter C_max_, t_max_, AUC 0–28 days, C_through_; treatment emergent adverse events; heart rate; body temperature; blood pressure; duration and morphology of P, QRS, and T waves in ECG; standard hematology lab assessment and chemistry; standard sensory and motor assessment of body and PNS and CNS	60/12 up to 52 weeks	NCT03499119 [31]
**1. Erenumab three doses adjusted by weight** **2. Placebo**	Recruiting	III	Episodic migraine with or without aura	6 to 11 Years; 12 to 17 years	Change from baseline in MMDs	456/12 weeks	NCT03836040 [32]
**1. Erenumab three doses adjusted by weight** **2. Placebo**	Recruiting	III	Chronic migraine	6 to 11 Years; 12 to 17 years	Change from baseline in MMDs	286/12 weeks	NCT03832998 [33]
**Eptinezumab adjusted by weight**	Recruiting	I	Migraine with or without aura	6 to 17 Years	Area under curve (AUC) (0–infinity) eptinezumab; C_max_ eptinezumab	32/20 weeks	NCT04537429 [37]
**Eptinezumab 100 mg IV or 300 mg IV adjusted by weight**	Recruiting	III	Migraine with or without aura and chronic migraine	6 to 17 Years	Number of participants with treatment-emergent adverse events	610/44 weeks	NCT05164172 [38]
**1. Eptinezumab (300 or 100 mg IV adjusted by weight)** **2. Placebo**	Recruiting	III	Chronic migraine	12 to 17 Years	Change from baseline in MMDs averaged over weeks 1–12	285/12 weeks	NCT04965675 [39]
**Fremanezumab dose adjusted by weight**	Recruiting	III; open label	Migraine with or without aura and chronic migraine	6 to 17 Years	Incidence of AEs; incidence of participants with clinically significant changes in laboratory values; incidence of abnormal ECG findings; incidence of abnormal vital signs; incidence of abnormal physical examination findings; suicidal ideation	550/56 weeks	NCT04530110 [36]
**1. Galcanezumab** **2. Placebo**	Recruiting	III	Migraine with or without aura	6 to 17 Years	Change from baseline in the number of monthly migraine headache days	325/3 months	NCT03432286 [34]
**1. Galcanezumab** **2. Placebo**	Recruiting	III	Chronic migraine	12 to 17 Years	Change from baseline in the number of monthly migraine headache days	300/3 months	NCT04616326 [35]
**1. OnabotulinumtoxinA** **2. Placebo**	Active, not recruiting	II	Chronic migraine	8 to 17 Years	Pain scores (intensity); change in duration of migraine episode; frequency of migraine; PedMIDAS scoring reduction; opioid consumption	26 patients/48 weeks	NCT03055767 [42]
**1. Rimegepant 75 or 50 mg** **2. Placebo**	Active, not yet recruiting	III	Episodic migraine with or without aura	6 to 17 Years	Change from baseline in the mean number of migraine days per month as measured over the 12-week double-blind phase	1100/12 weeks	NCT05156398 [40]
**1. Topiramate ER capsules** **2. Placebo**	Recruiting	IV	Migraine with or without aura	6 to 11 Years	Frequency of migraine attack per 28 days during the treatment phase.	162/28 days	NCT04050293 [43]
**1. Topiramate ER capsules** **2. Placebo**	Recruiting	IV	Migraine with or without aura	6 to 11 Years	Change from baseline (last 28 days run-in period) in the monthly number of headache days during the 8-week maintenance period based on the diary.	132/16 weeks	NCT04748601 [44]

AE, adverse events; CNS, central nervous system; ECG, electrocardiogram; ER, extended release; MMDs monthly migraine days; NA, not applicable; PedMIDAS, Pediatric Migraine Disability Assessment; PNS, peripheral nervous system.

Although no RCTs have been published on the anti-CGRP pathway in pediatric age, some anecdotical evidence has already been reported with monoclonal antibodies against CGRP or its receptor. In a multicenter, retrospective study, erenumab, galcanezumab, and fremanezumab were used as off-label treatment for 112 adolescent patients (mean age 15.9 years) with difficult-to-treat primary headache disorders. Among them, 94 patients (83.9%) had chronic migraine, 12 (10.7%) had new daily persistent headache, and six (5.4%) had post-traumatic headache. At the first follow-up visit, the authors reported that 29.5% of patients had a significant reduction in headache frequency compared to baseline (−2.0 days; 95%CI −0.8 to −3.2) and significant benefit/functional improvement as perceived by the patients. The most common side effects were injection site reactions (17.0%) and constipation (8.0%); five patients (4.5%) discontinued due to side effects [45]. In a more recent case series, six adolescent patients (age range 15–18) with episodic or chronic migraine were treated with erenumab. Two patients had a reduction in MMDs of at least 50%; one patient reported subjective improvement, while three patients were non-responders and discontinued after the first dose of treatment. Only one patient reported treatment-related constipation [46].

Among four other active studies (28.6%), one phase II study with completed recruitment compared onabotulinumtoxinA vs. placebo in pediatric patients (aged 8 to 17 years) with chronic migraine [42]. The first results on a small sample size (15 patients with chronic migraine), using 155 units at 31 injection sites in 3-month intervals and follow-up visits every 6 weeks, showed a statistically significant decrease in frequency and intensity of migraines compared to placebo, and no adverse effects related to treatment were reported [47]. A few additional studies on the use of onabotulinumtoxinA in the pediatric population have been reported. A recent RCT randomized 125 adolescent patients (age range 12–17) using different dosages of treatment or placebo (onabotulinumtoxinA 155 U, n = 45; onabotulinumtoxinA 74 U, n = 43; placebo, n = 37); 115 completed the study (92.0%). As all the treatments (including placebo) reduced the frequency of headache days after 3 months, with no significant differences between treatments, the study did not show efficacy (i.e., a change in the frequency of headache days from baseline compared to placebo). However, the tolerability was satisfactory, with only four patients discontinuing due to inefficacy. The most reported treatment-emergent adverse events were neck pain (n = 8), upper respiratory tract infection (n = 7), migraine (n = 5), and nasopharyngitis (n = 5) [48].

Previously published case series and retrospective analyses [48] of the off-label use of onabotulinumtoxinA (40 up to 215 IU) in adolescents (age range 8–18 years) with refractory CM suggested that onabotulinumtoxinA could provide subjective and clinical relief of symptoms, including reduction in headache frequency and severity. A complete list of these studies has been reported previously [48]. Therefore, results on the clinical benefits of onabotulinumtoxinA in pediatric patients with chronic migraine are discordant, and future trials enrolling larger numbers of patients and exploring the use of placebo are needed.

In 2014, the US Food and Drug Administration (FDA) approved topiramate for migraine treatment in pediatric patients aged 12 to 17 years, being the first and only FDA-approved medication for migraine prevention in adolescents. Although the CHAMP study failed to show any significant superiority of treatment with amitriptyline or topiramate compared to placebo, the efficacy and safety of topiramate have been assessed in previous RCTs. Topiramate was proven to be superior to placebo in reducing frequency of migraine attacks in an RCT pilot study that enrolled adolescents (12 to 17 years) and in a post hoc analysis of the three largest randomized, double-blind, placebo-controlled trials. Overall, the recommended dose is 2 mg/kg per day, but it is recommended to slowly titrate the dose over 8–12 weeks to avoid adverse events [23]. Doses of 100 and 200 mg/day have been demonstrated to effectively decrease the frequency of migraine headaches, with 100 mg/day benefits outweighing risks. Potential adverse effects include weight loss, sedation or cognitive depression, and paresthesia [49]. Additional active studies are evaluating two different topiramate extended-release drugs (two phase IV studies) vs. placebo [43,44] and alpha lipoic acid (ALA) vs. flunarizine as an active comparator (one phase IV open label study) [41] (Table 1).

ALA is an endogenous molecule with a role in various enzyme complexes in mitochondria, and therefore in energy metabolism [50]. ALA is used as nutraceutical in patients with migraine. Previous studies have evaluated the effectiveness and safety of ALA in adults with migraine [51], and one in the pediatric population that compared topiramate alone or topiramate co-administered with ALA [52]. The combination of topiramate (50 mg/day)/ALA (300 mg/day) was superior in reducing monthly migraine frequency and attack duration to topiramate or ALA only [52]. Overall, evidence on nutraceuticals for migraine prevention in the pediatric population is poor, and there are no reports in the above-mentioned guidelines [12]. However, bearing in mind the CHAMP study results, nutraceuticals offer a potential inexpensive and safe alternative or addition to pharmacologic preventative drugs, with a relatively low adverse effect profile. An overview of studies with nutraceuticals in the pediatric population is provided elsewhere [49,50]. An ongoing open-label study is currently assessing only dietary supplements (powder containing coenzyme q10, blueberries, black current, vitamins, and magnesium) in migraine patients aged 8 to 17 years [NCT01010711]. At present, there are no ongoing RCTs including verapamil, beta blockers, cyproheptadine, valproic acid, gabapentin, or venlafaxine. Previous evidence of these drugs in pediatric patients are reported in the above-mentioned guidelines [12].

### 3.3. Acute Treatments

Similar to ongoing trials with preventative treatments, almost half of the active studies on acute treatments (6 on 13 active studies) involve the novel drugs recently approved as acute treatment for migraine in adults, namely, anti-CGRP small molecules (ubrogepant and rimegepant) and the serotonin receptor 5-HT_1F_ agonist lasmiditan. Specific features of the studies, including primary endpoint, status, and age range, are reported in Table 2.

Lasmiditan (low, mid, or high dose) [56,57], rimegepant (50 or 75 mg) [59,60], and ubrogepant (low or high dose) [61,62] are being investigated in two paired phase III ongoing active studies, in particular, an open-label investigation to assess long-term safety (48–58 weeks) and a randomized compared to placebo trial to assess, as a primary endpoint, 2 h freedom from pain. All these studies plan to enroll a large sample size, ranging from 600 to 1646 patients (Table 2). Until present, no studies with the off-label use of gepants for acute treatment have been reported in the pediatric population. On the other hand, a phase I, open-label, two-cohort study assessed lasmiditan pharmacokinetics (PK) 24 h after exposure and tolerability in pediatric patients (aged 6–17 years) with migraine [64]. The study enrolled 18 patients subdivided in two cohorts (15 to ≤ 40 kg and > 40 to ≤ 55 kg, respectively) who received single oral doses of 100 or 200 mg of lasmiditan. One patient in the 200 mg cohort discontinued due to adverse events (i.e., lacrimation, ataxia, confusion state, attention disturbance, dizziness, fatigue, irritability, and nausea). Overall, the frequency and severity of adverse events experienced by eight patients (including somnolence, dizziness, and fatigue) were mild, and no severe adverse events were reported. The PK results supported the weight-based dosing of lasmiditan in the pediatric population [64]. No other studies for lasmiditan in the pediatric population have been reported thus far.

Other active studies are currently evaluating various drugs and formulations (Table 2). Four phase III studies are assessing a dry sumatriptan intranasal composition with placebo [63], the sphenopalatine ganglion (SPG) block with 2% lidocaine using a Q-tip applicator vs. IV prochlorperazine in an emergency setting [54] and intranasal lidocaine via a nasal mucosal atomizer vs. placebo [55] (Table 2). Overall, triptans are the most extensively studied acute medications in pediatric migraine. A systematic review of 21 prospective randomized controlled clinical trials (including 273 children and 7026 adolescents with migraine) detailed their effectiveness in pediatric populations; they are less effective in children than adults, and most of the initial pediatric RCTs produced negative results [24,49,65]. Liquid intranasal triptans (namely, sumatriptan, zolmitriptan, and almotriptan nasal spray [NS]) are the most investigated in children and adolescents, with sumatriptan NS approved by European Medicine Agency (EMA), whereas zolmitriptan, almotriptan, and rizatriptan NS are approved for patients aged 12 years and older by the FDA. An overview of studies with NS triptans in a pediatric population has been previously provided [65]. An ongoing phase III study with intranasal sumatriptan is testing a sumatriptan dry nasal powder composition already approved by the FDA for adults [66], but no studies have been published on its effectiveness or safety in the pediatric population to date.

Although there is some evidence in adults (achieved with various techniques, including intranasal lidocaine) [67], studies on SPG blockade in the pediatric population are still lacking [68]. In general, few reports have been published on peripheral nerve blocks with local anesthetics or devices in children [49]. In 2017, an abstract reported 133 SPG procedures performed in 85 patients aged 7–18 years as acute treatment for migraine, which decreased headache pain 10 min after the procedure with no complications [69]. Recently, a prospective case series evaluated the SPG block for the prevention of chronic daily headache in 17 adolescents, receiving multiple blocks using a medical device: a benefit on the Patient’s Global Impression of Change (PGIC) score after three months of treatment with no adverse events was reported [68]. However, there are no published placebo-controlled studies of SPG blocks in the pediatric population to date.

A phase I study is assessing dexamethasone 0.6 mg/kg IV vs. placebo in an emergency department (ED) setting in pediatric patients with migraine after parenteral administration of prochlorperazine or metoclopramide and diphenhydramine [53]. Although the efficacy of IV dexamethasone (as well as other corticosteroids) in children with acute headache has not been studied, a recent RCT controlled with placebo assessed the effect of oral dexamethasone for the prevention of migraine recurrence in pediatric patients presenting in ED. Of the 20 enrolled participants, 90.9% were satisfied with treatment at discharge/after 48 h and 81.8% were satisfied with their treatment at a 7-day follow-up [70]. In one retrospective pediatric study, steroid administration did not produce any differences in migraine recurrence rates at 48 and 72 h [71], whereas another retrospective study, assessing the effectiveness of different acute headache treatments in a pediatric ED, found that a small proportion of patients (6.3%) received a corticosteroid and 64% were discharged from ED [72].

Finally, one open-label study (with a non-applicable phase) is evaluating the role of propofol slow infusion in a pediatric ED [58]. Propofol is a fast-acting intravenous anesthetic agent, with sedation and anticonvulsant properties. Some studies have reported the use of subanesthetic dosing to treat severe migraine without causing deep sedation and related adverse events. An open-label, randomized trial enrolled 66 patients (aged 7–19 years) with acute migraine attacks presenting in an ED. Propofol was compared with a control group treated with ketorolac 0.5 mg/kg IV, diphenhydramine 1 mg/kg IV, or metoclopramide 0.1 mg/kg. The VAS reduction was not significantly different between groups, but the patients treated with propofol were less likely to experience rebound headaches [26]. A previous retrospective cohort study, comparing propofol with diphenhydramine, non-steroidal anti-inflammatory drugs, and prochlorperazine, included 15 pediatric patients. The propofol group achieved a significantly better reduction in VAS compared to controls [26]. At present, there are no RCTs ongoing including intravenous magnesium or cannabinoids. Previous evidence of these drugs in pediatric patients are reported in the above-mentioned guidelines [24].

## 4. Conclusions

In recent years, new acute and preventative treatments for migraine have been introduced, considerably increasing the possibility to achieve clinically significant responses to treatments, even in severe patients with chronic migraine and drug resistance. However, up until now, these promising results have been limited to adults. The CHAMP study showing that the efficacy of amitriptyline and topiramate does not differ from placebo, mainly due to the high placebo response in the pediatric population, highlighted a problem in this group of patients inherent to clinical investigations and the ensuing unmet need for effective treatments [18]. Appropriately designed clinical trials following the IHS ad hoc guidelines that consider clinical differences in migraine symptomatology and placebo response in pediatric age are necessary to maximize the success of the trials and standardize results. Furthermore, some non-pharmacological treatments, such as CBT and non-invasive neuromodulation with various devices, are currently being assessed in clinical trials, potentially increasing the number of therapeutic options. There are still no drugs available for children and adolescents with exclusive indications for migraine treatment. The results of ongoing clinical trials for preventative and acute medications are highly anticipated.

## Figures and Tables

**Table 2:** Ongoing studies on acute treatments in the pediatric population.

Treatments and Comparators	Status	Phase	Diagnosis	Age Range	Primary Endpoint	Planned Patients/Outcomes Time	Trial Number
**1. Dexamethasone 0.6 mg/kg IV** **2. Placebo**	Recruiting	I	Migraine	8 to 18 years	Incidence of relapse following discharge from the emergency department	116/7 days	NCT02903680 [53]
**1. Intranasal sphenopalatine block with 2% lidocaine** **2. Intravenous prochlorperazine**	Recruiting	III	Migraine	8 to 18 years	Time to headache resolution; length of stay in the emergency department	72/up to 6 h	NCT03984045 [54]
**1. Intranasal lidocaine** **2. Placebo**	Recruiting	III	Migraine	10 to 20 years	Change in pain score using NRS	50/5, 10, and 20 min	NCT03576820 [55]
**Lasmiditan**	Recruiting	III; open label	Migraine with and without aura	6 to 17 years	Percentage of TEAEs; percentage of participants with discontinuations due to AEs	1000/12 months	NCT04396574 [56]
**1. Lasmiditan low, mid, or high dose** **2. Placebo**	Recruiting	III	Migraine with and without aura	6 to 17 years	Percentage of participants with pain freedom (high dose)	1646/2 h	NCT04396236 [57]
**Propofol slow infusion**	Recruiting	NA; open label	Migraine	7 to 18 years	Reduction in pain score on the 0–10 Numeric Pain Rating scale	40/5 to 60 min	NCT02485418 [58]
**Rimegepant 50 or 75 mg**	Recruiting	III, Open label	Migraine with and without aura	6 to 17 years	The occurrence of treatment-emergent AEs; serious AEs	600/58 weeks	NCT04743141 [59]
**1. Rimegepant 50 or 75 mg** **2. Placebo**	Recruiting	III	Migraine with and without aura	6 to 17 years	Pain freedom using the number of patients reporting no pain	1440/2 h	NCT04649242 [60]
**Ubrogepant**	Not yet recruiting	III; open label	Migraine with and without aura	6 to 11 years; 12 to 17 years	Percentage of participants with AEs, with potentially clinically significant: ECG, vital sign parameters, lab values; suicidal ideation or suicidal behavior; changes from baseline in the Behavior Rating Inventory of Executive Function (BRIEF 2) questionnaire	1200/54 weeks	NCT05127954 [61]
**1. Ubrogepant low or high dose** **2. Placebo**	Not yet recruiting	III	Migraine with and without aura	6 to 11 years; 12 to 17 years	Percentage of participants with pain freedom at 2 hours after the initial dose	1059/2 to 24 h	NCT05125302 [62]
**1. Sumatriptan nasal powder** **2. Placebo**	Recruiting	III	Migraine with and without aura	12 to 17 years	Number of participants who were headache-pain free at 120 min after treatment	420/2 h	NCT03338920 [63]

AE, adverse events; ECG, electrocardiogram; IV, intravenous; NA, not applicable; NRS, numeric rating scale; TEAE, treatment-emergent adverse events.
